# Challenging the Classical View: Recognition of Identity and Expression as Integrated Processes

**DOI:** 10.3390/brainsci13020296

**Published:** 2023-02-10

**Authors:** Emily Schwartz, Kathryn O’Nell, Rebecca Saxe, Stefano Anzellotti

**Affiliations:** 1 Department of Psychology and Neuroscience, Boston College, Boston, MA 02467, USA; 2 Department of Psychological and Brain Sciences, Dartmouth College, Hanover, NH 03755, USA; 3 Department of Brain and Cognitive Sciences, Massachusetts Institute of Technology, Cambridge, MA 02139, USA

**Keywords:** face identity, facial expression, deep neural networks, face recognition, emotions

## Abstract

Recent neuroimaging evidence challenges the classical view that face identity and facial expression are processed by segregated neural pathways, showing that information about identity and expression are encoded within common brain regions. This article tests the hypothesis that integrated representations of identity and expression arise spontaneously within deep neural networks. A subset of the CelebA dataset is used to train a deep convolutional neural network (DCNN) to label face identity (chance = 0.06%, accuracy = 26.5%), and the FER2013 dataset is used to train a DCNN to label facial expression (chance = 14.2%, accuracy = 63.5%). The identity-trained and expression-trained networks each successfully transfer to labeling both face identity and facial expression on the Karolinska Directed Emotional Faces dataset. This study demonstrates that DCNNs trained to recognize face identity and DCNNs trained to recognize facial expression spontaneously develop representations of facial expression and face identity, respectively. Furthermore, a congruence coefficient analysis reveals that features distinguishing between identities and features distinguishing between expressions become increasingly orthogonal from layer to layer, suggesting that deep neural networks disentangle representational subspaces corresponding to different sources.

## 1. Introduction

The human ability to recognize face identity and facial expression is used as a compass to navigate the social environment [1]. Identity recognition enables us to acquire knowledge about specific individuals that we can retrieve in future encounters [2,3]. Expression recognition helps us to infer the emotional states of an individual [4,5,6] and predict their future actions and reactions. However, face identity and facial expression coexist within a face image. Information about each property needs to be extracted without being confused with the other.

The classical view on the recognition of face identity and facial expression proposes that identity and expression are processed by distinct pathways [2,7]. In this view, the pathway specialized for identity discards expression information, and the pathway specialized for expression discards identity information. With respect to the underlying neural mechanisms, it has been proposed [7] that face identity is recognized by a ventral temporal pathway, including the occipital face area (OFA) [8] and the fusiform face area (FFA) [9]. By contrast, facial expression is recognized by a lateral pathway [7], including the face-selective posterior superior temporal sulcus (fs–pSTS) [10].

In support of this view, several lines of evidence show that ventral occipitotemporal regions, such as OFA and FFA, play an important role in the recognition of face identity. Studies using fMRI adaptation show that changes in identity lead to greater release from adaptation than changes in viewpoint [11]. Research using multi-voxel pattern analysis (MVPA) found that identity information can be decoded from responses in OFA and FFA [12,13,14,15,16]. Structural connectivity measures reveal that congenital prosopagnosics (participants with congenital impairments for face recognition) present with reduced white matter tracts in the ventral occipitotemporal cortex [17].

Other evidence indicates that fs–pSTS is a key region for the recognition of facial expression. The fs–pSTS responds selectively to faces [18] and shows greater responses to moving faces than static faces [19]. Furthermore, videos of dynamic facial expressions do not evoke increased responses in OFA and FFA to the same degree as in fs–pSTS [19]. Additionally, the patterns of activity in this region encode information about the valence of facial expressions [20,21]. Finally, patients with pSTS damage have deficits for facial expression recognition [22], providing causal evidence in support of the involvement of pSTS in facial expression recognition.

Nevertheless, there is also evidence that weighs against this view of separate representational streams. Previous work noted the lack of strong evidence in support of the classical view [23]. In particular, while findings that support the classical view indicate that the lateral pathway plays a role in expression recognition, they do not rule out the possibility that the ventral pathway might also play a role [24]. In the same manner, findings that suggest the involvement of the ventral pathway in identity recognition do not rule out the possibility that the lateral pathway might contribute to identity recognition as well. Moreover, recent research directly shows that recognition of face identity and facial expression might be more integrated than previously thought. FMRI adaptation studies find release from adaptation for changes in facial expression in FFA [11]. Other work has shown that the valence of facial expression can be decoded from ventral temporal regions, including OFA and FFA [21,25]. Duchaine and Yovel [24] proposed a revised framework in which OFA and FFA are engaged in processing face shape, contributing to both face identity and facial expression recognition. At the same time, identity information can be decoded from fs–pSTS [26,27,28]. In fact, in one study, identity could be decoded with higher accuracy from fs–pSTS than from both OFA and FFA ([28], Figure 6), and two other studies demonstrated that identity could be decoded in fs–pSTS across faces and voices [26,27]. Furthermore, pSTS damage leads to impairments for recognizing face identity across different facial expressions [22], suggesting that pSTS plays a causal role for identity recognition as well. Finally, animal studies recently identified the middle dorsal face area (MD) in macaque monkeys. Interestingly, this face-selective area was shown to encode information on both face identity and facial expression [29]. Importantly, the area encodes identity robustly across changes in expression, and expression robustly across changes in identity [29], providing the strongest direct empirical challenge to the classical view.

The above evidence indicates that recognition of facial expression and face identity are implemented by integrated mechanisms, and not by separate neural pathways. Here, we offer a computational hypothesis that can account for this phenomenon. Unlike the classical view, which suggests that information relevant to identity recognition should be shed as representations of facial expressions develop, we hypothesize that representations optimized for expression recognition contribute to identity recognition and vice versa. Moreover, this occurs because identity and expression are entangled sources of information in a face image, and disentangling one helps to disentangle the other (the “Integrated Representation of Identity and Expression Hypothesis”—IRIEH).

IRIEH leads to two non-trivial computational predictions. First, if recognition of face identity and facial expression are mutually beneficial, training an algorithm to recognize face identity might lead to the spontaneous formation of representations that encode facial expression information and, likewise, training a separate algorithm to recognize facial expression might lead to the spontaneous emergence of representations that encode face identity information. Second, if this phenomenon occurs because disentangling identity from expression helps to also achieve the reverse, then integrated representations would not arise because recognition of identity and expression rely on common features. On the contrary, features important for the recognition of face identity and features important for the recognition of facial expression should become increasingly disentangled and orthogonal along the processing stream.

In the present article, we tested ‘in silico’ these computational hypotheses inspired by the neuroscience literature. To do this, we analyzed representations of face identity and facial expression learned by deep convolutional neural networks (DCNNs). DCNNs achieve remarkable accuracy in image recognition tasks [30,31], and features extracted from deep network layers have been successful at predicting responses to visual stimuli in the temporal cortex in humans [32] and in monkeys [33] (see [34,35] for reviews). Although artificially crafted stimuli (‘metamers’) have revealed differences between DCNNs and humans [36], DCNNs show similarities to human vision in terms of their robustness to image variation [37]. Recent work used DCNNs to test computational hypotheses of category-selectivity in the ventral temporal cortex [38]. In this article, we follow a similar approach and argue that a clearer understanding of representations of face identity and facial expression within DCNNs can serve as the foundation for future research on face representations in the brain.

To test our two predictions, we studied whether features from hidden layers of a DCNN trained to recognize face identity (from here onward the “identity network”) could be used successfully to recognize facial expression (see [39] for a related analysis). Symmetrically, we evaluated whether features from hidden layers of a DCNN trained to recognize facial expression (the “expression network”) could be used to identify face identity. In line with our anticipated results, we found that in a DCNN trained to label one property (i.e., expression), the readout performance of the non-trained property (i.e., identity) was not just preserved, but improved, from layer to layer. This was in stark contrast with classical theories of abstraction in visual processing that suggest that information about task-orthogonal information is progressively discarded [40,41,42]. Finally, we investigated the relationship between features encoding information that distinguish between identities and expressions across different layers of the DCNNs. We demonstrated that identity-discriminating features and expression-discriminating features became increasingly orthogonal over the network layers.

## 2. Materials and Methods

### 2.1. Stimuli

The identity network was trained to label identities using face images from the Large-Scale CelebFaces Attributes (CelebA) dataset [43]. CelebA is made up over 300,000 images. To match the dataset training size used for the expression network (see below), a subset of CelebA was used. The subset of the dataset contained 28,709 images for training and an additional 3589 images for testing (these latter images were used to test the performance of the network after training), and contained 1503 identities. These identities were randomly chosen, with at least 20 images per identity. All images were cropped to 178×178 pixels, resized to 48×48 pixels, and converted to grayscale by averaging pixel values of the red, green, and blue channels.

The expression network was trained to label facial expressions using the face images in the Facial Expression Recognition 2013 (FER2013) dataset [44]. The dataset contained 28,709 images for training and an additional 3589 images labeled as ‘public test’ (these latter images were used to test the performance of the network after training and to compare it to human performance). All images were originally sized 48×48 pixels and grayscale.

A network trained to recognize scenes was also implemented for comparison. The UC Merced Land Use dataset [45], which consisted of 2100 images of 21 classes, was used to train the network to label land images. All images were resized to 48×48 pixels and converted to grayscale by averaging pixel values of the red, green, and blue channels.

**Figure 1:**
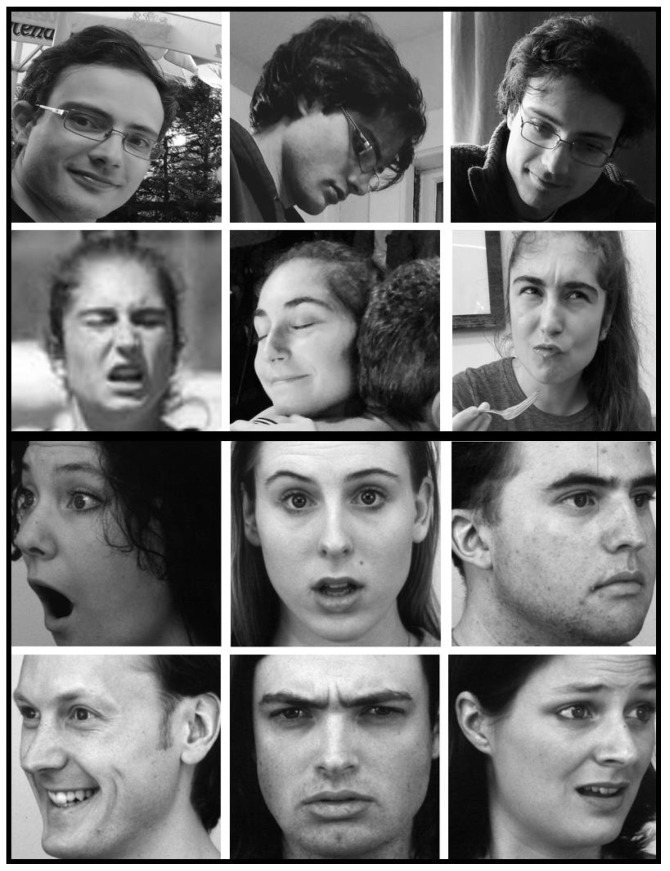
Face image examples. Top: naturalistic face images, similar to those from the CelebA and FER2013 datasets. Bottom: selected images from KDEF dataset (AF01AFHR, AF02SUHL, AF05AFS, AM01ANS, AM10HAHL, AM27NEHR).

The performance for each network was tested on stimuli from an independent dataset: the Karolinska Directed Emotional Faces (KDEF) dataset [46]. The KDEF dataset consisted of 4900 images depicting 70 individuals showing 7 different facial expressions from 5 different angles, each combination photographed twice. We used the frontal view images and those with views rotated by 45 degrees in both directions (left and right). Images were sized 562 (width) × 762 (height) and in color (RGB). For network transfer testing, in order to match the format of the training images, all KDEF images were converted to grayscale, cropped to squares, and downsampled to 48 × 48 pixels. The images were converted to grayscale by averaging pixel values of the red, green, and blue channels. As the positioning of the face within the image was consistent across KDEF images, the rectangular images were all cropped to the same 388 × 388 pixel region around the face. Example face images from the KDEF dataset, and example images similar (due to copyrights) to the CelebA and FER2013 datasets can be seen in Figure 1. Visual inspection confirmed that the face was visible in each KDEF image after cropping. Table 1 provides specific details about training and validation/testing set sizes.

### 2.2. Neural Network Architecture

**Figure 2:**
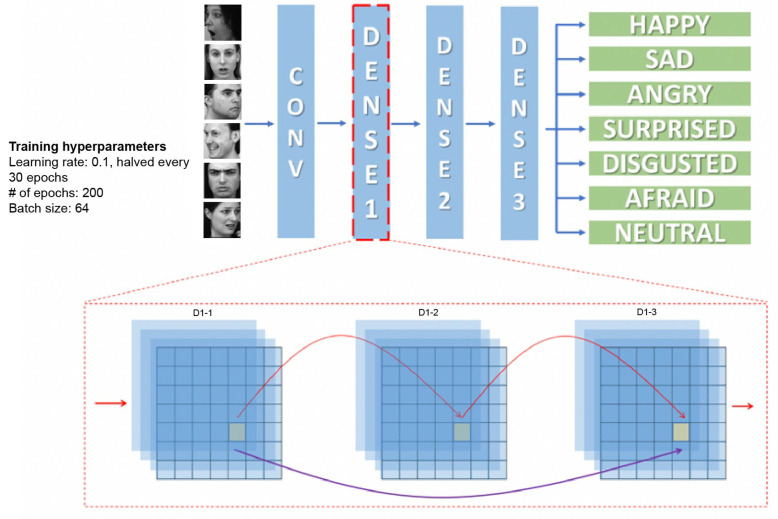
Neural network architecture. Top: Each network consists of a convolutional layer, three dense layers, and a linear classifier. Expression classification is used as an example here. Bottom: Single dense block; red arrows represent connections that would exist in a typical convolutional neural network, the purple arrow represents connections that are unique to the densely-connected network. Selected images from KDEF dataset: AF01AFHR, AF02SUHL, AF05AFS, AM01ANS, AM10HAHL, AM27NEHR.

Using Pytorch [47], a densely-connected deep convolutional neural network (DenseNet) was implemented, consisting of 1 convolutional layer, 3 dense blocks, and 1 fully connected linear layer (Figure 2). A DenseNet architecture was selected since it has been shown to yield high performance on a variety of tasks [48], and because it features connections between non-adjacent layers, bearing a closer resemblance to the organization of the primate visual system [49]. The convolutional layer consisted of 64 channels of 2D convolutions using a 3×3 kernel and padding = 1. Each dense block consisted of 3 densely connected convolutional layers with kernel size = 3, stride = 1, and padding = 1. Each layer in the dense block produced 32 channels of output. Therefore, the number of input channels for the first layer in a dense block was equal to the number of output channels of the previous layer outside the dense block (i.e., for the first layer of the first dense block it was equal to 64: the number of output channels of the first convolutional layer). The number of input channels for each subsequent layer in each dense block increased by 32. This choice is widely used and featured on publicly available DenseNet implementations (i.e., https://github.com/pytorch/vision/blob/master/torchvision/models/densenet.py, accessed on 1 November 2019).

Each dense block (except the last) was followed by a transition layer that received, as input, the outputs from all layers of the dense block plus the layer preceding the dense block, and produced an output with half the number of channels using a max pooling with a 2×2 kernel. The last dense block was followed by an average pooling with an 8×8 kernel and then by a fully connected linear layer. In sum, the number of input and output channels for the 13 layers of the network can be seen in Table 2.

All layers used rectified linear units (ReLU) as nonlinearity for an activation function. All layers in the dense blocks and all transition layers used 2D dropout with a dropout probability p=0.1 [50]. All convolutional layers were followed by batch normalization [51].

### 2.3. Training and Validation

We evaluated 4 sets of networks: identity-trained, expression-trained, scene-trained, and untrained (randomly initialized weights). Each network described was implemented 10 times with random weight initialization to test the consistency of the results. We report the average accuracy across the 10 initializations, including the standard error of the mean in the figures as error bars.

Given 48 × 48 grayscale images in the CelebA dataset, the identity network was trained to recognize 1503 face identities varying in pose and age. The network was trained to minimize the cross-entropy loss between the outputs and true labels using stochastic gradient descent. The learning rate began at 0.1 and halved every 30 epochs. The training was run for 200 epochs, and images were presented to the network in batches of 64. The performance of the trained network was validated using an independent subset of CelebA that was not used for any of the training. The identity network labeled face identity with an accuracy of 26.5% on the held-out ‘test’ images (chance performance at 0.06%). The CelebA database did not include viewpoint labels, so we were unable to test cross-viewpoint validation performance.

The expression network produced an output of 7 values, one for each expression label in the dataset (surprised, angry, fearful, disgusted, sad, neutral, and happy). The network was trained to minimize the cross-entropy loss between the output and the true labels using stochastic gradient descent, with a learning rate starting at 0.1 and halved every 30 epochs. The training was run for 200 epochs, and images were presented to the network in batches of 64. After training, the accuracy of the expression network was validated using an independent subset of the FER2013 dataset that was not used for training (the images marked as ‘PublicTest’). The network achieved an accuracy of 63.5% (chance performance at 14.2%), closely matching the reported human accuracy on the FER2013 stimuli (65%) [44]. The FER2013 database did not include viewpoint labels, so we were unable to test cross-viewpoint validation performance.

The scene network was trained to recognize various land images. This network matched the architecture used for the identity and expression networks, and followed the same training and validation protocols. The trained network was able to label the validation set with an accuracy of 80.95%. The untrained network (with randomly initialized weights) used the same architecture as all other networks, but it did not undergo any training.

### 2.4. Transferring to KDEF

After training with each dataset was completed, the weights of each network were fixed (‘frozen’) to prevent further learning. Henceforth, we refer to a network that has the weights fixed after the initial training as a ‘pre-trained network’. To test identity and expression labeling, we used a new dataset of images: the KDEF dataset [46], in which each image has both an identity and an expression label.

#### 2.4.1. Labeling Identity across Expression and Expression across Identity

To evaluate whether the identity network could successfully perform the task it was trained for, we tested whether it could accurately label identity in the KDEF dataset. Then, we tested the identity network’s performance at labeling expression. To assess the transformation of representations across different stages of the neural network, we evaluated the readout accuracy of identity and expression for features extracted from different layers. For each of the 10 identity networks trained with the CelebA dataset, accuracy was evaluated for features extracted from the first convolutional layer, and for features extracted from the last layer in each dense block, after they had been summed with the inputs of the block. The outputs that the networks needed to produce for identity labeling and for expression labeling were different. For instance, the number of identity labels was different than the number of expression labels (70 v 7). To accommodate for this, we extracted the corresponding layer feature representations by running an image through the pre-trained model (up until the specified layer). We then ran the image’s feature representation through batch normalization, ReLU, and an average pooling with an 8×8 kernel, followed by a fully connected linear layer that produced, as output, the identity or expression labels (referred to as the ‘readout layer’ from here on). Critically, these added fully connected readout layers achieved very different performances depending on the layer of the network that they were attached to (that is, depending on the nonlinear features that they received as an input). Readout performance was then tested on the held-out portion of the KDEF data. The performance of a linear layer trained directly on pixel values was used as a control.

We followed an analogous procedure for the expression network. First, we tested the expression network to ensure that it could accurately perform the expression recognition task on the KDEF dataset. Next, for each of the 10 expression networks, we used the same readout procedure as above to probe the accuracy of expression and identity labeling. To assess the transformation of representations across different stages of the neural network, accuracy was evaluated for features extracted from the first convolutional layer, and the last layer in each of the 3 dense blocks, after they had been summed with the inputs of the block. As in the case of the identity network, the performance of a linear layer trained directly on pixel values was used as a control.

Due to the ability of these models to rely on low-level features, we partitioned the KDEF dataset into training and testing sets, and tested the models across different viewpoints. To look at cross-viewpoint generalization, the identity and expression networks’ performances were tested with a readout layer trained using all but one of the viewpoints (frontal, 45 degree left, or 45 degree right), and accuracy was tested using the held-out viewpoint (as in [14]). Accuracy values for both identity and expression labeling were then averaged across the three conditions. This choice was made to provide a more stringent test of identity and expression recognition, as rotation in depth alters all parts of the face.

The added readout layers’ performances were heavily dependent on the nonlinear features received as inputs. If the added readout layers trained with a subset of the KDEF images could achieve high accuracy without needing the features from a pre-trained network, this should have been evident when they were attached to early layers of that pre-trained network (or when attached to layers of the untrained network, see below). When using features from late layers as compared to features from early layers of the pre-trained networks, accuracy improvements could not be due to the attached readout layer that was trained with a subset of KDEF images because the same readout layer was used for both early and late layers.

#### 2.4.2. Labeling Identity and Expression Using Untrained and Scene Network Features

The procedure described above was enacted to evaluate the performance of identity and expression labeling on KDEF images using the following: (1) randomly initialized, untrained neural network weights and (2) scene-optimized neural network weights. KDEF images were run through the various networks and their feature representations were extracted at multiple layers. The same readout procedure was used to learn the identity and expression labels for the KDEF images. After training the readout layer only, identity and expression labeling performances on the various KDEF feature representations were obtained.

### 2.5. Overlap between Identity and Expression Features

If, as we predicted, information about the non-trained feature (i.e., identity for the expression network and expression for the identity network) was not discarded during training, there were two potential explanations. First, it could be that the same image features were important for classifying both identity and expression. Alternately, it could be that distinct image features were important for classifying identity and expression, and both were retained within the network. In this case, the presence of features that contributed to labeling the irrelevant task indicated that the abstraction-based model of feature representations in the brain was not supported by the kind of representations that were learned spontaneously by the deep convolutional neural networks. In order to dissociate these outcomes, we tested the congruence of the spaces spanned by the opposing identity and expression features in all 3 of the trained (identity, expression, and scene) networks.

To do this, we averaged a layer’s responses across different expressions, obtaining an average response pattern across the layer features for each identity. Next, we used principal component analysis (PCA) to extract the 5 dimensions that explained most of the variation across identities. The same procedure was repeated by averaging layer responses across identities, obtaining an average response pattern for each expression, and ultimately 5 dimensions that explained most of the variation across expressions.

Finally, we used a congruence coefficient (introduced in [52]) to evaluate the similarity between the spaces spanned by the features. Considering the matrix $L_{e}$ of the loadings of principal components for expression on the layer features and the matrix $L_{i}$ of the loadings of principal components for identity, we obtained the matrix $S=L_{i}L_{e}^{\prime }L_{e}L_{i}^{\prime }$ and measured overlap as the sum of the eigenvalues of *S*, which was equal to the sum of the squares of the cosines of the angles between all pairs of principal components where one component in the pair was for expression and the other was for identity [52].

**Figure 3:**
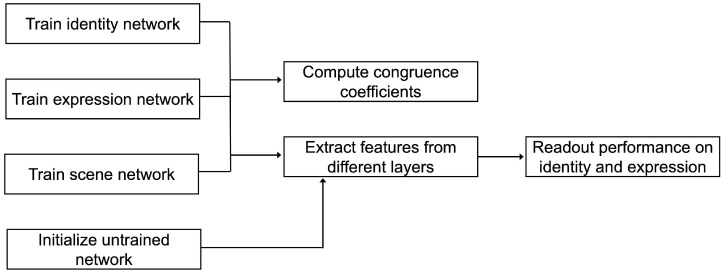
Analysis flowchart. An overview of the analysis steps performed.

An overview representing the research procedure can be seen in Figure 3.

## 3. Results

### 3.1. Validation Performances of Trained Neural Networks

A densely-connected deep convolutional neural network (DenseNet, [48], Figure 2) was trained to recognize face identity using a subset of the CelebA dataset. The network was able to label face identity with an accuracy of 26.5% on the held-out ‘test’ images (chance performance at 0.06%). A confusion matrix can be found in Supplementary Materials (Figure S1A).

A DenseNet ([48], Figure 2) was trained to recognize facial expressions (surprised, angry, fearful, disgusted, sad, neutral, happy) using over 28,000 facial expression images (FER2013). The network was able to label facial expression on the held-out ‘test’ images with an accuracy of 63.5% (chance performance at 14.2%). A confusion matrix can be found in Supplementary Materials (Figure S1B).

A third DenseNet ([48], Figure 2) was trained to label land images. The network was able to label the different scene categories on the held-out ‘test’ images with an accuracy of 80.95% (chance performance at 4.76%). A confusion matrix can be found in Supplementary Materials (Figure S1C).

### 3.2. Neural Networks Trained to Recognize Identity Develop Expression Representations

Recognition of face identity across changes in viewpoint is notoriously difficult [14,53]. Thus, we aimed to investigate the invariance of the identity network’s face representations across image transformations. To do this, we used images from the KDEF dataset that included frontal views, as well as 45 degree views (left and right) of the faces. We explored, across different viewpoints, whether the identity network could label both face identity and facial expression after the newly attached readout layer was trained using two of the three views, and then, tested with the held-out view.

**Figure 4:**
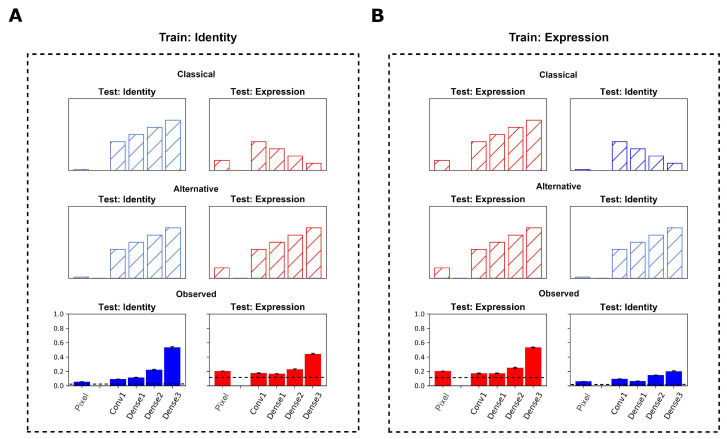
Identity and Expression Networks. (**A**) Identity Network. (**Top** row) Expected pattern of results following a classical view of abstraction. (**Middle** row) Expected pattern of results following an alternative view of abstraction. (**Bottom** row) Observed Results. Classification accuracy for identity (**left**) and expression (**right**) for a readout layer attached to successive sections of the pre-trained identity network. Dotted line represents performance at chance. Leftmost bar represents performance of the unattached linear classifier. (**B**) Expression Network. (**Top** row) Expected pattern of results following a classical view of abstraction. (**Middle** row) Expected pattern of results following an alternative view of abstraction. (**Bottom** row) Observed Results. Classification accuracy for expression (**left**) and identity (**right**) for a readout layer attached to successive sections of the pre-trained expression network. Dotted line represents performance at chance. Leftmost bar in each plot represents performance of the unattached linear classifier. Error bars denote the SEM of the performance of each network instance.

The identity network generalized to the KDEF dataset for identity recognition. The network achieved an accuracy of 53.82% (chance performance at 1.42%) when testing on held-out viewpoints (Figure 4A, bottom left). The readout layers that received the identity network’s extracted features as inputs achieved a higher accuracy for identity recognition when testing on a held-out viewpoint, compared to a fully connected linear layer that received pixel values of the KDEF images as inputs. Specifically, the linear layer that received the pixel values as inputs achieved an accuracy of 6.31%. By contrast, readout layers applied to the features from the convolutional layer, first, and second dense blocks yielded accuracy values of 9.61%, 11.91%, and 22.65% respectively (Figure 4A, bottom left). Thus, accuracy increased from layer to layer.

Having established that the identity network successfully generalized to the KDEF dataset for the task it was trained to perform (identity recognition), we next studied whether the identity network developed features that could yield accurate expression recognition when testing on the held-out viewpoint. As detailed in the Methods section, in order to generate the 7 facial expressions as output (instead of the 70 face identity labels), a readout layer was attached to the outputs of a hidden layer of the pre-trained identity network, and then trained with KDEF images consisting of two viewpoints to label expression. Critically, the identity network weights were fixed at this stage, and only the weights of the newly attached readout layer would be able to change.

When using identity-trained weights, expression classification of images from the KDEF dataset across different viewpoints (44.37%, Figure 4A, bottom right) was greater than chance. By contrast, a linear layer that received pixels as inputs achieved an accuracy of 20.40%. Importantly, as in the case of identity classification, the accuracy of the network increased from early layers to late layers. Readouts of features extracted from the initial convolutional layer, and first and second dense blocks of the identity network yielded accuracy values of 17.61%, 16.67%, and 23.02%, respectively, when labeling expression, finally reaching 44.37% in the third dense block, as mentioned previously (Figure 4A, bottom right). A large increase in accuracy was observed in the second and third dense blocks, paralleling the increase in accuracy observed for identity labeling at the same processing stages. This indicated that in the network trained to label identity and then tested on expression recognition, the findings deviated from the predictions of the classical view (Figure 4A, top right).

### 3.3. Neural Networks Trained to Recognize Expression Develop Identity Representations

In parallel to the identity network analysis, we investigated the invariance of the expression network’s face representations across image transformations. The expression network was not trained to recognize identity across different viewpoints, but it was trained to label facial expression across viewpoints. Could the features it developed for labeling facial expression be used to support the demanding task of view-invariant identity recognition? To address this question, we again used images from the KDEF dataset showing a frontal view as well as 45 degree views (left and right) of the faces. We investigated whether the expression network could label facial expressions and identities when the newly attached readout layer was trained with two of the three views, and then tested with the held-out view.

The final accuracy at cross-viewpoint expression labeling on the KDEF images was high (53.43%, Figure 4B, bottom left), showing that the expression network generalized successfully to the new dataset. As expected, labeling accuracy increased from layer to layer of the expression network. A readout layer applied directly to the pixels of the KDEF images obtained an accuracy of 20.40% for expression classification, but subsequent layers were necessary to reach the final accuracy of 53.43%. Features extracted from the initial convolutional layer, and first and second dense blocks of the expression network yielded accuracy values of 17.22%, 17.31%, and 24.93%, respectively, when labeling expression (Figure 4B, bottom left). Similar to the patterns in accuracy that were found when using the identity network, a large increase in accuracy was observed in the third dense block with a final accuracy of 53.43% (Figure 4B, bottom left).

Next, the expression network weights were used to label identity. In order to generate the 70 identities as output (instead of the 7 facial expression labels), a readout layer was attached to the outputs of a hidden layer of the expression network pre-trained with the FER2013 dataset, and trained with images consisting of 2 viewpoints to label identity. The expression network weights were fixed at this stage, and only the weights of the newly attached readout layer could change.

Final identity classification of images from the KDEF dataset (20.2%, Figure 4B, bottom right) was greater than chance. By contrast, linear classification using the pixels as input achieved an accuracy of only 6.31%. Importantly, readout accuracy increased from early to late layers in the network. Features extracted from the initial convolutional layer, and first and second dense blocks of the expression network, yielded accuracy values of 9.56%, 6.32%, and 14.81%, respectively, when labeling identity, reaching a final accuracy of 20.20% in the third dense block (Figure 4B, bottom right). An increase in accuracy was observed in the second and third dense blocks. Although to a smaller degree, this paralleled the increases in accuracy observed for expression labeling at the same processing stages. This finding was in contrast with the decrease in identity information that would have been expected in the classical view (Figure 4B, top right).

### 3.4. Recognition of Identity and Expression Using Features from an Untrained Neural Network

We next aimed to investigate an untrained network’s face representations across image transformations. Like before, we used images from the KDEF dataset showing a frontal view as well as 45 degree views (left and right) of the faces. We explored whether the randomly initialized, untrained network could label facial expressions and face identities when the newly attached readout layer was trained with two of the three views, and then tested with the held-out view.

**Figure 5:**
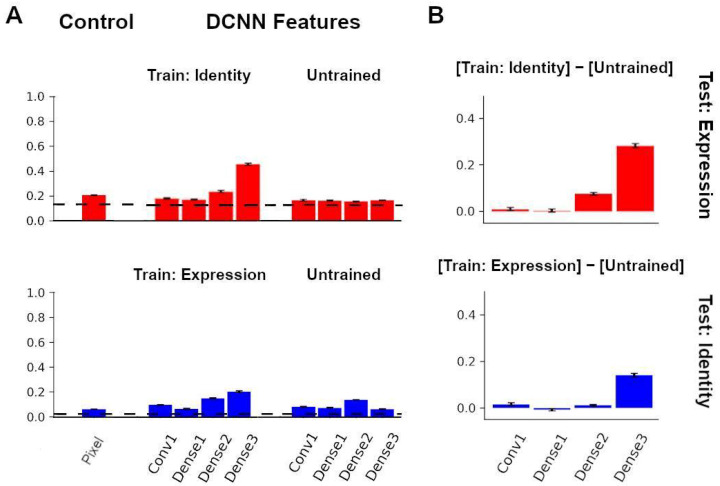
Comparisons with the Untrained Network. (**A**) Classification performance using identity features and untrained features for expression labeling (**top**) and expression features and untrained features for identity labeling (**bottom**). (**B**) Difference in expression classification between identity network and untrained network (**top**). Difference in identity classification between expression network and untrained network (**bottom**). Error bars in plots denote the SEM of the performance of network instances.

For expression labeling, features extracted from the initial convolutional layer, and the first, second, and third dense blocks of the untrained network yielded accuracy values of 16.54%, 16.22%, 15.51%, and 16.51%, respectively (Figure 5A, top right). The untrained network performed similarly for all layers of the network, with each layer performing close to chance level.

For identity labeling, features extracted from the initial convolutional layer, and the first, second, and third dense blocks of the untrained network yielded accuracy values of 7.90%, 7.13%, 13.62%, and 6.10%, respectively (Figure 5A, bottom right). The untrained network decreased in classification performance overall.

Figure 5B shows the accuracy differences for expression and identity labeling when subtracting the untrained network performance from the trained network performance of the transferred task. Overall, the difference between the transferred task performance and the untrained performance increased from layer to layer, showing the relative advantage of the trained network.

### 3.5. Recognition of Identity and Expression Using Features from a Neural Network Trained to Recognize Scenes

To test the transfer performance of a network trained to recognize an unrelated category, we explored the ability of a network trained for scene recognition to label facial expression and face identity across image transformations. Unlike facial expression and face identity recognition tasks, which both involve face images as inputs, scene recognition does not involve faces. We examined whether a scene network (that received no face input during training) could label facial expression and face identity after the newly attached readout layer was trained using two of the three views, and was then tested with the held-out view from the KDEF dataset.

**Figure 6:**
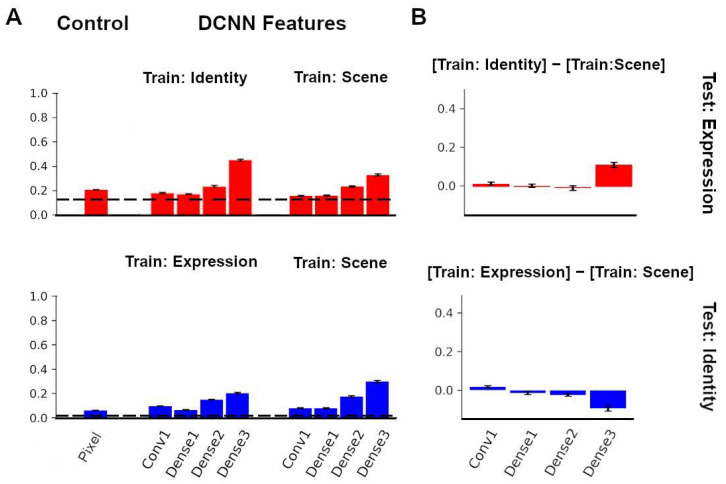
Comparisons with the Scene Network. (**A**) Classification performance using identity features and scene features for expression labeling (**top**) and expression features and scene features for identity labeling (**bottom**). (**B**) Difference in expression classification between identity network and scene network (**top**). Difference in identity classification between expression network and scene network (**bottom**). Error bars in plots denote the SEM of the performance of network instances.

When labeling expression, features extracted from the initial convolutional layer and first, second, and third dense blocks of the scene network yielded accuracy values of 15.9%, 16.0%, 23.5%, and 33.0%, respectively (Figure 6A, top right). Although the scene network increased from layer to layer, it did not perform as well as the expression and identity networks for expression classification. The differences in accuracy between the identity and scene network for expression labeling can be seen in Figure 6B (top).

When labeling identity, features extracted from the initial convolutional layer, and the first, second, and third dense blocks of the scene network yielded accuracy values of 9.5%, 7.8%, 17.3%, and 29.6%, respectively (Figure 6A, bottom right). Although the scene network increased from layer to layer, it did not perform as well as the identity network. However, interestingly, the scene network was more accurate at identity labeling than the expression network. This can be seen in Figure 6B (bottom).

### 3.6. Overlap between Identity and Expression Features May Decline across Layers

Different hypotheses could account for the observed increase in accuracy for identity labeling in correspondence with the increase in accuracy for expression labeling. According to one hypothesis, recognition of face identity and facial expression might rely on similar features. Therefore, the features learned by the network trained to recognize expression would also yield good accuracy when labeling face identity. Instead, according to a different hypothesis, recognizing identity and expression would require disentangling two generative sources that jointly contribute to the same image. In this case, separating what aspects of the image were due to identity could prevent a neural network from erroneously attributing those aspects to expression. For this reason, a neural network trained to label identity or expression might develop representations of expression and identity, respectively. The representations could then be used to disentangle identity and expression, even when recognition of identity did not rely on the same features as expression recognition.

If the features that were most useful for labeling identity and expression were similar, the dimensions that best discriminated between identities and those that best discriminated between expressions should also be similar. Thus, the angles between identity dimensions and expression dimensions should be small and congruence should be high. If, on the other hand, features needed to recognize identity and expression were disentangled by the net, the angles between identity dimensions and expression dimensions should become increasingly larger from layer to layer. Furthermore, if training with identity or with expression induced disentanglement between identity and expression features, training the network with scene images should yield comparatively higher congruence between identity and expression features compared to training with identity or expression.

**Figure 7:**
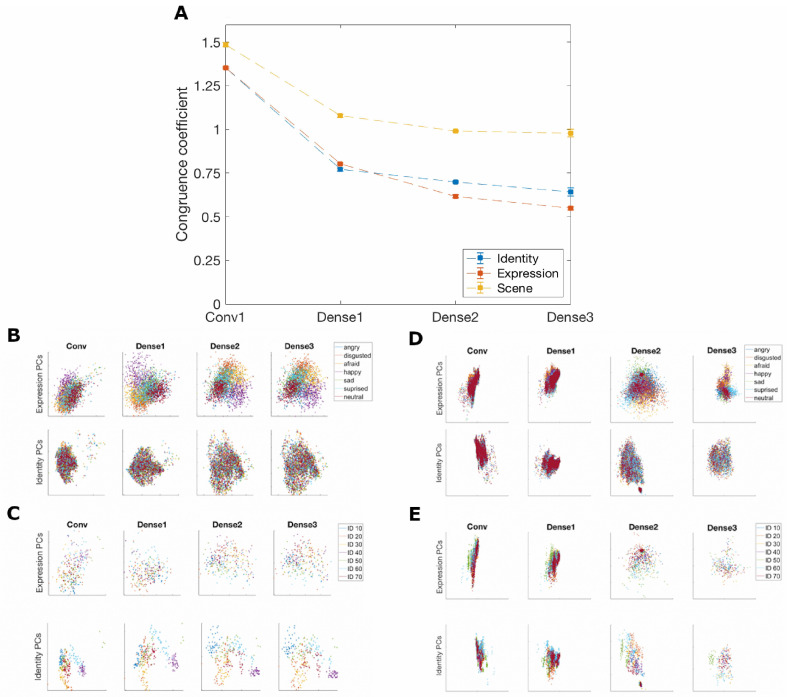
Trained neural networks and principal components. (**A**) Identity, expression, and scene network congruence coefficients between principal components derived from activations averaged over expression and identity. (**B**) Face activations labeled by expression projected into expression and identity principal component spaces for each layer of the identity network. (**C**) Face activations labeled by identity (only 7 of 70 identities are displayed for clarity) projected into expression and identity principal component spaces for each layer of the identity network. (**D**) Face activations labeled by expression projected into expression and identity principal component spaces for each layer of the expression network. (**E**) Face activations labeled by identity (only 7 of 70 identities are displayed for clarity) projected into expression and identity principal component spaces for each layer of the expression network.

We differentiated between these predictions by calculating a congruence coefficient between the first five principal components (PCs) for expression and the first five PCs for identity for each layer of each trained neural network. A larger congruence coefficient would signify that the identity and expression dimensions were more similar to one another, and a smaller congruence coefficient would indicate they were less similar. In both the network trained to label identities and the network trained to label expressions, the PCs for identity and expression exhibited higher congruence values in the earliest layer. For both the identity and expression networks, congruence decreased from layer to layer (Figure 7A). The scene network’s congruence values followed the same decreasing pattern. However, the congruence coefficients between identity and expression were larger compared to the other networks, indicating that the identity and expression features were less disentangled in the scene network.

For visualization purposes, the activation patterns across network features in response to different face images were projected onto the top two identity and expression PCs for each layer within a network (see Figure 7B–E). In each case, the relevant aspect (expression or identity) visibly clustered in deeper layers of the net, while the other aspect did not, further showing that discrimination of expression and identity relied on co-existing but different features.

## 4. Discussion

Recent studies revealed the presence of information about face identity and facial expression within common brain regions [26,28], challenging the view that recognition of face identity and facial expression are implemented by separate neural mechanisms, and supporting alternative theoretical proposals (i.e., [24,54]). In the present study, we proposed the Integrated Representation of Identity and Expression Hypothesis (IRIEH), according to which recognition of face identity and facial expression are ‘complementary’ tasks, such that representations optimized to recognize face identity also contribute to the recognition of facial expression, and vice versa. This would account for the observation that both identity and expression information coexist within common brain regions, including the face-selective pSTS [26,28]. Based on IRIEH, we predicted that features from artificial deep networks trained to recognize face identity would be able to support accurate recognition of facial expression, and reciprocally so too would features from deep networks trained to recognize facial expression be able to support accurate recognition of face identity.

To evaluate this hypothesis, we trained a deep convolutional neural network (DCNN) to label face identity, and found that, as the labeling of identity increased in accuracy from layer to layer, the labeling of expression also correspondingly improved, despite the fact that the features of the identity network were never explicitly trained for expression recognition. We also demonstrated that this phenomenon was symmetrical. The same DCNN architecture trained to label expression learned features that contributed to labeling identity, even though the features of the expression network were never explicitly trained for identity recognition. Additionally, in the models that we tested, features from a network trained to categorize scenes also supported identity and expression recognition, indicating that this phenomenon might not be restricted to within domain-tasks.

Our findings could serve as proof, that in order to perform identity recognition, expression information does not necessarily need to be discarded (and vice versa). In fact, within the set of models that we tested in this article, networks trained to perform one task did not just retain information that could be used to solve the other task, but rather, they enhanced it. The accuracy for labeling expression achieved with features from intermediate layers of the network was higher than the accuracy achieved with features from early layers. Likewise, the accuracy of labeling identity using features trained for expression recognition improved over layer progression. These same patterns held for the identity network, in that accuracy improved over the layers when labeling identity and expression.

In seeming contrast with our results, a previous study [55] found that features became increasingly specialized for the trained task in the later layers of the network. In the present article, despite features encoding expression and identity becoming increasingly orthogonal from early to late layers, accuracy at labeling progressively increased for the tasks. A fundamental difference that sets apart the study by Yosinski and colleagues [55] from the present study is that we attached a read-out layer directly to the frozen hidden layer, rather than continuing to train the rest of the model. When retraining multiple layers, starting from an early pre-trained layer yields better accuracy [55]. However, our results indicated that, at least in the case of identity and expression, when using a simple readout, features from later layers yielded better accuracy than features from earlier layers.

Lastly, one could conclude that the increase in performance seen in late layers was not due to common features found between tasks. Our factor congruence analysis comparing identity and expression spaces suggested that the similarity between the dimensions that best distinguished between identities and the dimensions that best distinguished between expressions decreased from layer to layer in both the identity and expression networks (and this was true for the identity and expression dimensions from the scene network as well). Since a small amount of congruence remained, it was not possible to rule out some overlap. However, the representations of identity and expression became increasingly orthogonal from layer to layer. Our findings dovetailed with previous work that proposed that object recognition was a process of untangling object manifolds [56,57]. Each image of an object can be thought of as a point in a high-dimensional feature space, and an object manifold is the collection of the points corresponding to all possible images of an object. Using pixels as the features, object manifolds are not linearly separable. Object recognition maps images onto new features that make the object manifolds linearly separable [56]. In the case of face perception, we can think of face identity manifolds (the points corresponding to all possible images of a given face identity), and facial expression manifolds (the points for all images of a given expression). By interpreting the identity and expression results from this perspective, face perception is not only limited to untangling identity manifolds, but also to untangling expression manifolds. In other words, the process of untangling one set of manifolds naturally untangles the other to some extent, similar to pulling two ends of yarn to unravel a knot.

There are several aspects that need to be taken into consideration when interpreting our findings. First, while our results do provide a proof of principle that identity representations arise naturally in simple, feedforward architectures trained to achieve near-human accuracy at expression recognition and vice versa, this does not guarantee that all neural network architectures show the same effect. Nevertheless, in support of the view that recognition of identity and expressions might be more integrated than previously thought, some recent studies tested one direction of this classification (training on identity and testing on expression) for the top layers of a ResNet-101 [39] model and a VGG-16 [58] model, providing some converging evidence that this phenomenon is not restricted to the one specific neural network architecture.

Secondly, although DCNNs share similarities with brain processing, findings from DCNN models cannot be directly used to reach conclusions about the human brain [59]. Nonetheless, DCNNs are a useful tool for proof of principle tests of computational hypotheses (see [60] for an elegant example) and can inspire us to generate hypotheses that we can then test with neural data.

Finally, we found that while untrained DCNNs did not lead to increasing accuracy for identity and expression recognition from layer to layer, transfer from DCNNs trained for scene recognition to face tasks (identity and expression) performed similarly to transfer from DCNNs trained for one of the face tasks (e.g., identity) to the other face task (e.g., expression). Thus, our findings cannot be interpreted as supporting the possibility that face-selectivity in the brain might be the result of greater transfer accuracy for tasks within a same category (e.g., faces) than across categories. Note that each network was retrained ten times to account for random variation in weight initialization, indicating that these results were consistent across multiple choices of the networks’ initial weights.

Given the scene network’s transferring ability, an open question that remains is why a model that was trained to recognize scenes was able to label identity and expression with increasing performance. Substantial evidence indicates that face and scene processing are specialized tasks and do not take place within the same brain regions [7,61]. If the DCNN models show that shared representations for scenes and faces are possible, then why does this not occur in the brain? One can speculate that there may be other mechanisms that may constrain category-specificity [38]. For instance, one can envision this using different types of neural network modeling, such as models that leverage multi-task learning. If one were to train a multi-task neural network to perform identity and expression recognition together and a different multi-task neural network to perform identity and scene recognition simultaneously, the former may perform significantly better than the latter. Taken together, it is likely that different sets of algorithmic learning principles determine the constraints of category-specificity.

## 5. Conclusions and Future Directions

This article demonstrates the spontaneous emergence of representations of facial expression when deep neural networks are trained to label face identity, as well as the spontaneous emergence of representations of face identity when deep neural networks are trained to label facial expression. Similar phenomena might occur in other domains. One study reported related evidence for the emergence of representations of viewpoint and position in the visual field for deep networks trained to label objects [62]. In addition, late layers of deep networks trained to recognize identity encode information about yaw and pitch [63]. Table 3 shows studies that similarly examined network transfer learning abilities to other tasks. More broadly, integrated implementation of complementary computations might be a large-scale principle of the organization of the human cortex, determining by virtue of computational efficiency, what sets of cognitive processes are represented within the same neural systems. As such, complementarity could apply to cases as diverse as word recognition and speaker recognition in speech processing, syntax and semantics in language, and the inference of mental states and traits in social cognition. This proposal is broadly related to the idea of a taxonomy of tasks (‘Taskonomy’ [64,65]), which might not be restricted to the domain of vision.

Future work can test whether face representations generated for labeling expression or identity also support recognition of the other feature with invariance across different kinds of transformations, such as translation, scaling, and the more challenging case of occlusion. We would also expect DCNNs trained to recognize expression and identity to encode information about other properties of faces, such as age, sex, race, pitch, and yaw. In addition, facial expressions are dynamic, and extending the present results to neural networks processing dynamic stimuli will be an important step forward to better understand the relationship between features built for expression recognition and features built for identity recognition.

## Figures and Tables

**Table 1:** Dataset information.

Dataset	Training Set Size	Testing/Validation Set Size	Stimulus Type
CelebA [43] ^1^	28,709	3589	Face
FER2013 [44]	28,709	3589	Face
UC Merced Land Use [45]	1890	210	Scene
KDEF [46]	2520–2646 ^2^	294–420 ^2^	Face

^1^ Only a subset of the CelebA dataset was used to train and test the identity model. ^2^ Number of images used for training and held-out for testing depended on labeling task.

**Table 2:** Hyperparameters of the networks’ layers.

Layer Name	Kernel Size	Input Channels	Output Channels
Conv1	3 × 3	1	64
Dense1-1	3 × 3	64	32
Dense1-2	3 × 3	96	32
Dense1-3	3 × 3	128	32
Transition1	2 × 2	160	80
Dense2-1	3 × 3	80	32
Dense2-2	3 × 3	112	32
Dense2-3	3 × 3	144	32
Transition2	2 × 2	176	88
Dense3-1	3 × 3	88	32
Dense3-2	3 × 3	120	32
Dense3-3	3 × 3	152	32
Average pooling	8 × 8	152	32
Fully Connected	1 × 1	32	1

**Table 3:** Comparison of studies evaluating different DCNN generalizations.

Study	Identity -> Expression	Expression -> Identity	Object Category -> Category-Orthogonal Properties
Current Study	X	X	
Colón et al. (2021) [39]	X		
Hong et al. (2016) [58]			X
Zhou et al. (2022) [62]	X		

## Data Availability

Code for training and testing the neural networks, as well as feature extraction can be found at: https://github.com/els615/3DenseNets. Image data was obtained from four online databases. CelebA is available at: https://mmlab.ie.cuhk.edu.hk/projects/CelebA.html [43], accessed on 1 February 2021. FER2013 is available at: https://www.kaggle.com/datasets/msambare/fer2013 [44], accessed on 1 November 2019. UC Merced Land Use is available at: http://weegee.vision.ucmerced.edu/datasets/landuse.html [45], accessed on 1 July 2021. KDEF is available at [46].

## Supplementary Materials

The following supporting information can be downloaded at: https://www.mdpi.com/article/10.3390/brainsci13020296/s1, Figure S1: Confusion matrices.
