# Supplementary figures and images for: Challenging the Classical View: Recognition of Identity and Expression as Integrated Processes
Source: Brain Sci. 2023 Feb 10;13(2):296. doi: 10.3390/brainsci13020296 (PMC9954353; doi:10.3390/brainsci13020296)

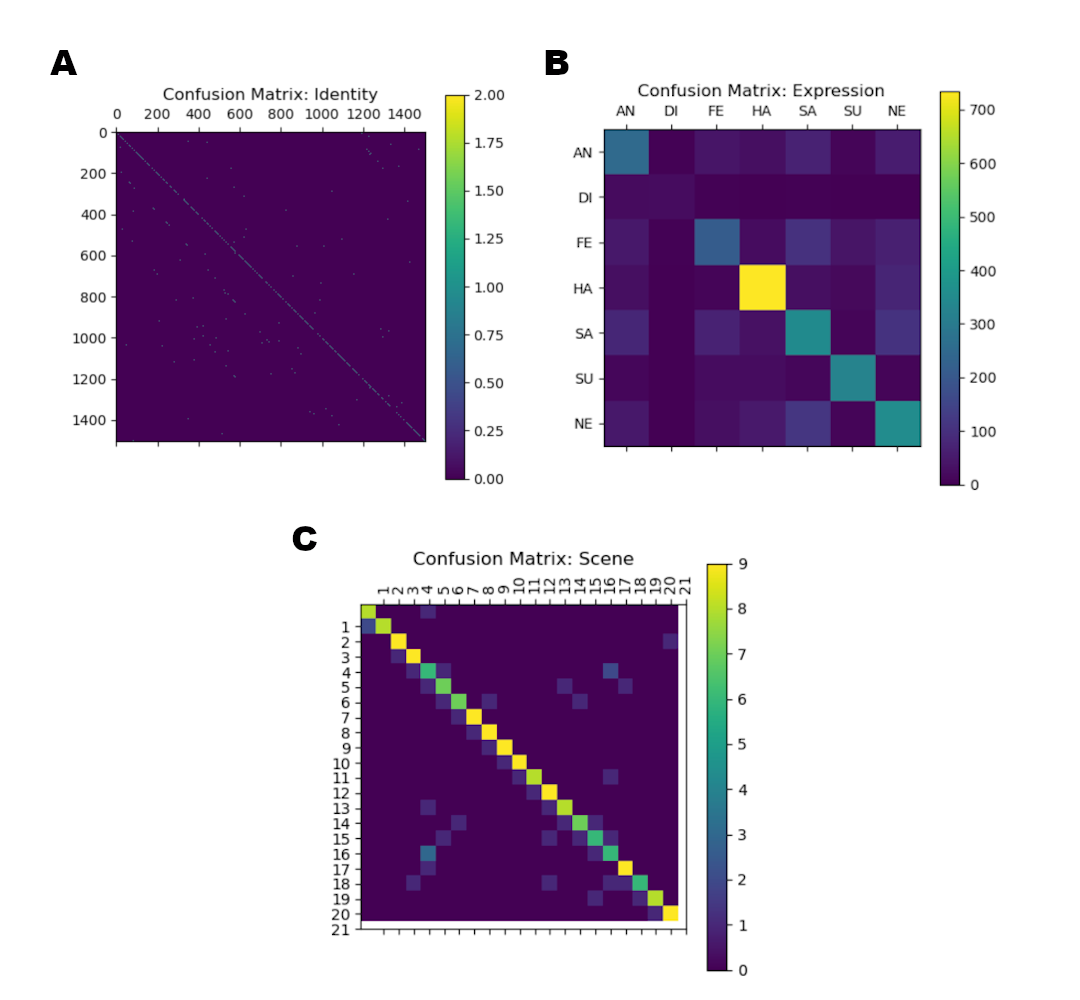

Supplement: Supplementary file 1 [file brainsci-13-00296-s001.zip › brainsci-2143079-supplementary.png]
